# Structural Organization and Function of the Golgi Ribbon During Cell Division

**DOI:** 10.3389/fcell.2022.925228

**Published:** 2022-06-24

**Authors:** Inmaculada Ayala, Antonino Colanzi

**Affiliations:** Institute of Experimental Endocrinology and Oncology “G Salvatore” (IEOS), National Research Council (CNR), Napoli, Italy

**Keywords:** Golgi ribbon, mitosis, spindle, cancer, checkpoint

## Abstract

The Golgi complex has a central role in the secretory traffic. In vertebrate cells it is generally organized in polarized stacks of cisternae that are laterally connected by membranous tubules, forming a structure known as Golgi ribbon. The steady state ribbon arrangement results from a dynamic equilibrium between formation and cleavage of the membrane tubules connecting the stacks. This balance is of great physiological relevance as the unlinking of the ribbon during G2 is required for mitotic entry. A block of this process induces a potent G2 arrest of the cell cycle, indicating that a mitotic “Golgi checkpoint” controls the correct pre-mitotic segregation of the Golgi ribbon. Then, after mitosis onset, the Golgi stacks undergo an extensive disassembly, which is necessary for proper spindle formation. Notably, several Golgi-associated proteins acquire new roles in spindle formation and mitotic progression during mitosis. Here we summarize the current knowledge about the basic principle of the Golgi architecture and its functional relationship with cell division to highlight crucial aspects that need to be addressed to help us understand the physiological significance of the ribbon and the pathological implications of alterations of this organization.

## Introduction

The Golgi complex (GC) is a central organelle of the secretory pathway (Glick and Nakano, 2009) and is also an important intracellular signaling platform (Luini and Parashuraman, 2016). In the majority of eukaryotes, the GC is organized in the form of stacks of cisternae that are functionally polarized, each containing a distinct set of cargo-processing enzymes (Wei and Seemann, 2010). The proteins and lipids synthesized in the endoplasmic reticulum (ER) are transported to the *cis-*Golgi through an intermediate compartment (IC) that is considered a stable sorting station between the ER and the GC (Appenzeller-Herzog and Hauri, 2006). The cargoes are then processed to be finally sorted at the trans-Golgi network (TGN) for the transport to specific plasma membrane location or other organelles (Glick and Nakano, 2009). Recent evidence suggests that the TGN is an organelle independent of the GC (Nakano, 2022) and that it operates in close functional and spatial connection with the recycling endosomes (RE), where endocytosed materials travel before recycling to the plasma membrane (Fujii et al., 2020).

In addition, in most vertebrate cells, adjacent stacks are connected by complex tubular-saccular membranous structures, the “non-compact zones” (Rambourg and Clermont, 1990), to form a continuous system (Cole et al., 1996) called Golgi ribbon, generally located near the centrosome (CE) (Wei and Seemann, 2010). If biological evolution has favored this organization, it should have an advantage for the cells. Yet, the functional role of the ribbon is still not completely understood, as the isolated stacks are fully capable of performing basic tasks of transport and cargo modifications (Wei and Seemann, 2010). The current evidence suggests that the ribbon organization could increase the efficiency of the processing, transport and polarized delivery of selected cargo, and regulate signaling events (Makhoul et al., 2018; Li et al., 2019a; Kulkarni-Gosavi et al., 2019; Ravichandran et al., 2020).

**FIGURE 1:**
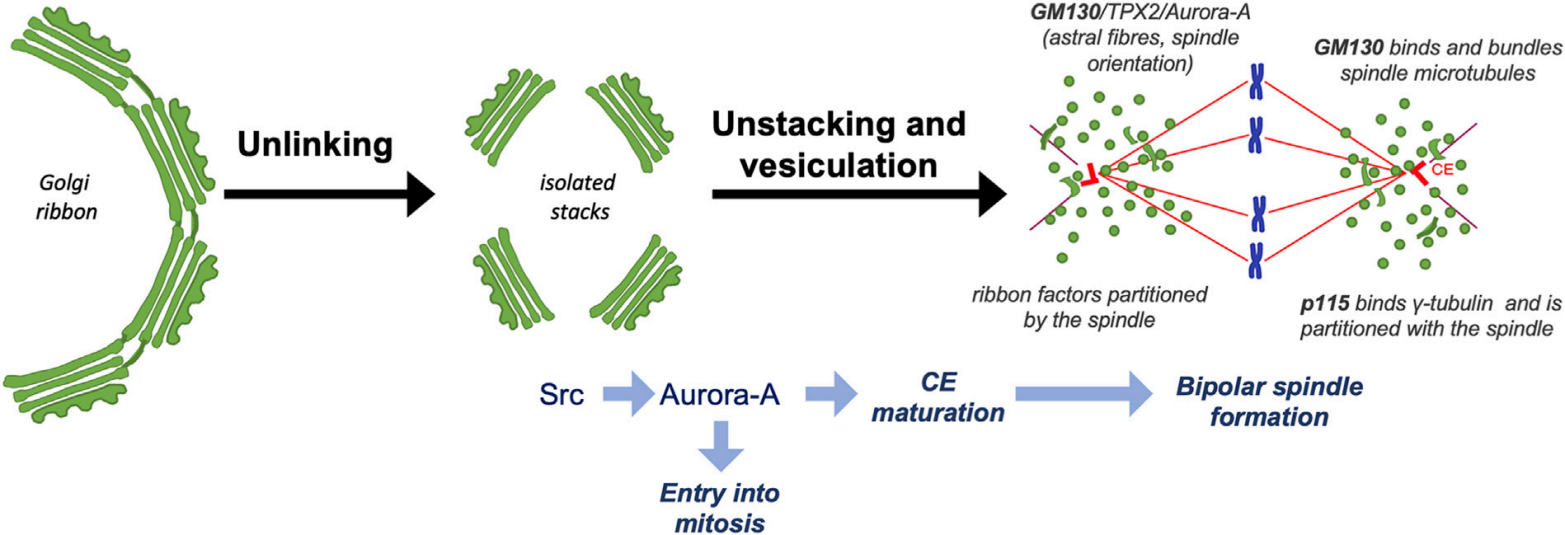
Schematic description of GC disassembly during G2 and functional connections with mitosis. During G2, the GC is unlinked into stacks, leading to the activation of a Src/Aurora-A signaling axis that drives centrosome maturation and entry into mitosis. After mitosis onset, the Golgi stacks are disassembled into dispersed vesicles and clusters. Golgi-associated proteins, including GM130 and p115, are repurposed to form a bipolar, symmetric and correctly oriented spindle that mediates the inheritance of proteins necessary for GC ribbon reformation at mitotic exit.

Several reports have shown that during G2 the Golgi ribbon must be unlinked into the constituent stacks to allow entry into mitosis (Figure 1) (Colanzi et al., 2007; Feinstein and Linstedt, 2007). Accordingly, blocking the unlinking step results in a potent G2-block of the cell cycle, pointing out that a “Golgi checkpoint” oversees the correct pre-mitotic cleavage of the GC (Sutterlin et al., 2002; Hidalgo Carcedo et al., 2004), and revealing that the cell cycle progression depends not only on the checkpoints that supervise DNA replication and spindle formation, but also the segregation of the GC. Similarly, a role for other organelles in regulating mitosis-specific events is also emerging (Mascanzoni et al., 2019).

Although the crucial role of the GC in driving cell division is well established, there are many details about the mechanisms and regulation of ribbon formation and the control of mitotic events that are only partially understood. In addition, there are general questions that need to be addressed. For example, why is ribbon cleavage required for entry into mitosis? How accurate must the partitioning of Golgi proteins be? In this article we summarize the major features of the mechanism of GC maintenance, referring to other reviews for more details, and present our view about why the answers to these questions could help us to understand the physiological significance of the ribbon structure and the pathological implications of alterations of this organization.

### Structural Organization of the Golgi Complex

The isolation of a “Golgi matrix” more than 25 years ago allowed the identification of a set of proteins with fundamental roles in Golgi structure, transport and function (Slusarewicz et al., 1994). The golgins are major components of this matrix, comprising at least 11 members that are associated with the Golgi membrane through a variety of mechanisms (Gillingham and Munro, 2016). The golgins are characterized by an elongated structure composed of coiled-coil domains that can be extended into the cytoplasm for up to 200 nm. They are involved in vesicle tethering, interaction with the cytoskeleton, Golgi enzyme retention and scaffolding for signaling complexes (Lowe, 2019). The various golgins are localized to distinct domains of the GC, in agreement with their functional specialization (Gillingham and Munro, 2016; Wong et al., 2017).

Additional components of the matrix are the Golgi Reassembly and Stacking Proteins (GRASPs; GRASP65 (Barr et al., 1997) and GRASP55 (Shorter et al., 1999)). They are peripheral proteins associated with the Golgi membrane via an N-terminal myristoylation. GRASP65 forms a complex with the golgin GM130 at the *cis*-Golgi (Barr et al., 1998; Hu et al., 2015), whereas GRASP55 binds Golgin 45 (Short et al., 2001; Zhao et al., 2017). Although GRASP55 is commonly considered a medial-localized protein, a recent super-resolution imaging approach led to the conclusion that both GRASPs reside at the *cis*-Golgi (Tie et al., 2018). GRASP65 and GRASP55 have a conserved N-terminal “GRASP domain” composed of two PDZ domains (Vinke et al., 2011; Li et al., 2019a) that can homodimerize in *trans*. Several biochemical and structural data suggest that the GRASP homodimers tether adjacent membranes, thus guiding the juxtaposition of cisternae to form the stacks or the fusion of homologous cisternae of adjacent stacks to form the ribbon (Zhang and Wang, 2015; Rabouille and Linstedt, 2016). The homodimerization is inhibited by phosphorylation of residues present in the C-terminal regulatory regions, offering a mechanism for the regulation of the structure of the GC (Ayala et al., 2020). GRASP65 and GRASP55 are also involved in a variety of non-structural functions, as they interact with a set of cargo, cargo adaptors and glycosylation enzymes, thereby regulating the intra-Golgi traffic of proteins and glycosphingolipid biosynthesis (Rabouille and Linstedt, 2016). They are also differentially involved in signaling as GRASP65 plays a crucial role in apoptosis, while GRASP55 is involved in Golgi stress response, unconventional protein secretion and autophagy (Zhang and Wang, 2020).

The structure and function of the GC are under the control of GTPases and several signaling pathways, including those involved in phosphoinositide metabolism and mitogen-activated protein kinase signaling (Kulkarni-Gosavi et al., 2019). Moreover, clustering and position of the stacks depend on the concerted actions of CE- and Golgi-nucleated microtubules (MTs) and actin cytoskeleton (Ravichandran et al., 2020; Mascanzoni et al., 2022). Despite this complexity, the GC can rapidly modify structure and localization in response to specific physiological conditions (Nakamura et al., 2012; Gosavi and Gleeson, 2017; Saraste and Prydz, 2019; Ravichandran et al., 2020).

### The Ribbon is Actively Separated Into Stacks During G2

At steady state, the GC structure results from the dynamic equilibrium between the formation and cleavage of the membranes connecting the stacks (Colanzi et al., 2007). Live imaging showed that ribbon formation is mediated by the elongation of GRASP-coated membranous tubules from the cisternae of “donor” stacks (Feinstein and Linstedt, 2008; Cervigni et al., 2015). The elongation of these tubules requires phospholipases and is driven by dynein and MTs (Bechler et al., 2012). Eventually, the tubules dock and fuse with homologous cisternae of “acceptor” stacks (Feinstein and Linstedt, 2008). The essential role of GRASPs in ribbon formation is supported by several loss-of-function experiments based on siRNA-mediated depletion, functional inactivation through kinase inhibitors, and microinjection of blocking antibodies or recombinant proteins (Valente and Colanzi, 2015; Rabouille and Linstedt, 2016; Zhang and Wang, 2020). Moreover, experimental approaches designed to induce rapid degradation of both GRASPs showed that acute depletion of either GRASP unlinks the Golgi ribbon (Burd, 2021). Specifically, GRASP65 degradation shows a prevalent loss of continuity at the *cis*-side, whereas downregulation of GRASP55 generally affects the continuity at the medial/*trans* level (Jarvela and Linstedt, 2014). Of note, rapid degradation of both GRASPs does not induce evident unstacking (Grond et al., 2020; Zhang and Seemann, 2021), thus questioning the role of GRASPs in stacking. A possible explanation is that stacking is provided by redundant mechanisms that could temporarily compensate for the loss of GRASPs.

Among the proteins involved in ribbon formation, a key role has emerged for the GM130/GRASP65 complex. Indeed, GM130 recruits the scaffold AKAP450, which drives the nucleation of GC-originated MTs that are crucial for the clustering of the stacks (Rivero et al., 2009; Sanders and Kaverina, 2015; Ravichandran et al., 2020). In addition, GRASP65 stabilizes Golgi-associated MTs, favoring ribbon formation (Ayala et al., 2019), in agreement with tomography data showing that MTs are preferentially associated with the *cis*-side of the GC (Marsh et al., 2001). The C-terminal domain of GRASP65 binds the DnaJ homolog subfamily A member 1 (DJA1), which promotes GRASP65 oligomerization (Li et al., 2019b) and the actin regulator Mena, which drives the formation of short actin fibers (Tang et al., 2016), probably involved in stabilizing the inter-stack connections. Thus, the GM130/GRASP65 complex coordinates several membrane-based events and the cytoskeleton to allow the formation of the lateral bridges connecting the stacks. Whether similar mechanisms also involve GRASP55 is not known. The inverse process of cleavage of the connections is driven by the fission-inducing protein BARS, but the mechanism and regulation are not known (Figure 1) (Hidalgo Carcedo et al., 2004; Colanzi et al., 2007). The only regulatory mechanism known to date is the inhibition of the tethering step. Specifically, during G2 the kinase PKD activates the RAF1/MEK1/ERK1 signaling cascade that phosphorylates GRASP55 (Acharya et al., 1998; Colanzi et al., 2003; Feinstein and Linstedt, 2008; Kienzle et al., 2013), while JNK2 and PLK1 phosphorylate GRASP65 (Sengupta and Linstedt, 2010; Cervigni et al., 2015) (Figure 1). These phosphorylations concur to block tethering events, resulting in Golgi unlinking (Rabouille and Linstedt, 2016; Wei and Seemann, 2017).

Hence, the current evidence indicates that the GRASPs are major effectors of signaling pathways regulating the ribbon organization. Nevertheless, critical aspects of ribbon formation/disassembly are unclear, including the “trigger” of the signaling acting on the GRASPs, the potential role of additional regulatory pathways, the role and regulation of the golgins, and the mechanism and regulation of BARS, the cytoskeleton and membrane fusion-based processes. The investigation of these aspects is of paramount importance for a global understanding of the regulation and maintenance of the Golgi ribbon and for developing approaches for its modulation.

An additional area that is poorly understood is related to the precise structure of the inter-stack connections. Indeed, even if the ribbon organization appears to be mediated by the formation and fusion of tubules connecting the stacks (Feinstein and Linstedt, 2008), the few ultrastructural studies conducted so far indicate that the non-compact zones have the form of tubular-saccular membranes, suggesting that these membranes have a structure more complex than that of simple tubules (Saraste and Prydz, 2019). Therefore, we hope for a revived interest in this topic thanks to the development of electron microcopy (EM) approaches characterized by improved resolution and automatization (FIB-SEM, STEM, Array Tomography) (Ferguson et al., 2017; Koga et al., 2017; Ohta et al., 2021) that now also allow a faster examination of the detailed ultrastructure of larger and deeper Golgi areas.

### Why is Golgi Unlinking Required for Entry Into Mitosis?

The identification of the “Golgi mitotic checkpoint” opened the question of how the segregation of this organelle controls entry into mitosis. Related to this issue, we have shown that the unlinking activates a specific Golgi-associated pool of Src, which then interacts with the mitotic kinase Aurora-A. The consequent Src-mediated phosphorylation of Y148 of Aurora-A results in increased activity and centrosome-recruitment of Aurora-A, which then triggers entry into mitosis and CE maturation (Figure 1) (Barretta et al., 2016), a prerequisite for spindle formation (Marumoto et al., 2002; Hoar et al., 2007; Malumbres and Perez de Castro, 2014). Whether Golgi unlinking controls entry into mitosis only through the Src-Aurora-A signaling axis is unclear.

Thus, the pre-mitotic segregation of the ribbon into stacks generates a signaling that is directly connected with those controlling cell duplication. Yet, a still open question is why Golgi unlinking is necessary for entry into mitosis. As the GC is closely associated with the CEs, a possibility is that Golgi unlinking could be necessary to remove a steric hindrance that could hamper CE separation in G2. However, this is unlikely since the GC is dissociated from the CE during this phase (Frye et al., 2020). Moreover, inhibition of Golgi unlinking does not alter CEs separation (Persico et al., 2010).

A hint could derive from the mitotic fate of Golgi membranes. After mitosis onset, CDK1 phosphorylates various golgins, GRASPs and adaptor proteins involved in mediating membrane fusion (Wang and Seemann, 2011). Consequently, based on *in vitro* reconstitution of Golgi fragmentation, it has been concluded that the stacks are disassembled through an extensive vesiculation process (Uchiyama et al., 2003; Kaneko et al., 2010; Tang and Wang, 2013; Totsukawa et al., 2013). CDK1 also induces the disassembly of ER exit sites, inducing a block of secretion. Correlated to this block, it has been shown that GFP-tagged Golgi enzymes cycling between the ER and the GC remain trapped in the ER during mitosis (Zaal et al., 1999). However, two different studies based on drug-induced ER fragmentation or retention of Golgi enzymes in the ER, reached the opposite conclusion that the Golgi proteins diffuse independently of the ER (Axelsson and Warren, 2004; Pecot and Malhotra, 2006). Yet, all these studies were based on the investigation of GFP-tagged Golgi markers. Thus, we think that it would be important to dedicate more efforts in investigating by immuno-EM the subcellular localization of several endogenous Golgi markers to have a broader and quantitative view of the process.

Independently on the mechanism, extensive disassembly is a peculiarity of the GC (Mascanzoni et al., 2019) and is crucial for mitotic progression, as blocking stacks disassembly induces the formation of monopolar spindles, resulting in the spindle checkpoint activation and arrest in metaphase (Guizzunti and Seemann, 2016).

The disassembly process is also associated with the change of localization of a set of Golgi-associated proteins that acquire new mitosis-specific functions. A significant example is offered by GM130, which possesses a nuclear localization signal that during interphase is masked by the interaction with the matrix protein p115 (Wei et al., 2015). CDK1-mediated phosphorylation of GM130 dissociates p115, allowing the nuclear localization signal to activate a signaling axis composed of the spindle assembly factor TPX2, which binds to and activates Aurora-A to control the formation of “astral” MTs, which are necessary for correct spindle orientation (Figure 1) (Wei et al., 2015; Guo et al., 2021). Moreover, GM130 can also stabilize the spindle fibers by direct binding to MTs, thus linking Golgi membranes to the spindle (Wei et al., 2015; Wei and Seemann, 2017). In agreement with this role, GM130 depletion causes the formation of multipolar mitotic spindles (Kodani and Sutterlin, 2008). The golgin p115 has an armadillo-like fold that mediates the interaction with γ-tubulin and localization at the CEs (Radulescu et al., 2011). Strikingly, depletion of either p115 or GRASP65 causes spindle abnormalities and defects in chromosome segregation (Figure 1) (Sutterlin et al., 2005; Radulescu et al., 2011). The mitotic spindle has also an active role in the mitotic inheritance of Golgi structural proteins as it mediates the partition of “ribbon factors” necessary for post-mitotic reassembly of Golgi ribbons (Figure 1) (Seemann et al., 2002; Wei and Seemann, 2009). Proteomic and fractionation experiments suggest that these “ribbon factors” include at least GM130, p115 and Golgin 160 (Sauer et al., 2005; Radulescu et al., 2011).

Therefore, we hypothesize that the Golgi checkpoint monitors ribbon separation in G2 because the unlinking process could be necessary to form two equivalent Golgi membrane pools as a prerequisite for the balanced redistribution at the spindle fibers of a set of GC-associated proteins. Their correct distribution could have two purposes: The first is to concur to form a symmetric bipolar spindle. The second is to allow the spindle-mediated partition of a minimal set of GC-associated scaffolds acting as templates for the reformation of two equivalent Golgi ribbons at mitotic exit (Figure 1) (Wei and Seemann, 2009; Ravichandran et al., 2019).

### Is the Equal Distribution of Golgi Membranes Important for Cell Duplication?

As the GC is a single copy organelle, it is evident that a certain degree of disassembly is necessary to produce two pools of membranes distributed in the daughter cells, thus allowing rapid GC reformation at the mitotic exit, as its *de novo* reformation would require several hours (Tangemo et al., 2011).

A related important question is how accurate the partitioning of Golgi enzymes and matrix proteins must be. At metaphase, before GC reassembly and reformation into the two daughter cells, the mitotic Golgi membranes are distributed into two different pools. One is enriched in Golgi enzymes characterized by rapid and MT-dependent diffusion and redistribution between individual vesicles or clusters dispersed throughout the cytoplasm (Axelsson and Warren, 2004). The second is enriched in clusters containing matrix proteins, mainly distributed around the spindle poles (Seemann et al., 2002).

The accuracy of the distribution of dispersed GC membranes has been examined in a limited number of studies based on confocal imaging, which led to the conclusion that the GC is partitioned through a mechanism more precise than a stochastic division (Shima et al., 1998). However, the fraction of Golgi membranes present in detectable clusters is a minor fraction of the total (Puri et al., 2004), suggesting that the majority of Golgi enzymes is contained in structures whose dimension is below the resolution limits of fluorescence microscopy, undermining the accuracy of the quantitative assessment of their distribution.

Additional aspects that merit further investigation are the dynamic of Golgi proteins and the composition of the Golgi clusters. Indeed, live imaging studies showed that the IC is not disassembled during mitosis, forming two pools of membranes associated with the CEs and spindles (Marie et al., 2012). These studies also led to the conclusion that the mitotic IC clusters are intermediate stations of vesicle-mediated ribbon and stacks disassembly and are in a bidirectional exchange of material with the Golgi “haze,” suggesting that the IC could represent a template of GC reformation at the mitotic exit (Marie et al., 2012). Moreover, very little is known about the inheritance of the TGN.

Thus, until now, despite the several immunofluorescence (IF) and EM studies conducted in the 80s and 90s (Wang and Seemann, 2011), we still have an incomplete picture of the structure, distribution and dynamics of Golgi membranes during mitosis. Hence, this is an additional area of uncertainty that we think requires further investigation and that could benefit from the technological advances in the field of high-resolution imaging approaches (e.g., STED, SIM, STORM) (Feng et al., 2018). These approaches, coupled to the use of fluorescently labeled nanobodies or Fab fragments (Yang et al., 2022), could now allow a more quantitative detection of the location of Golgi proteins during mitosis. As an example, the development of dedicated computational analysis of images acquired through Airyscan and 3D-structured illumination microscopy, and the 4Pi single-molecule switching super-resolution microscopy, enabled the imaging of a large number of Golgi markers with a resolution of 5–10 nm (Zhang et al., 2020; Tie et al., 2022). Also, these techniques could be associated to correlative light and EM (CLEM) procedures, which are used to align fluorescent signals with EM imaging (Rizzo et al., 2014), to allow the high-resolution study of areas of interest selected by conventional fluorescence microscopy thus, to reveal the precise ultrastructure and distribution of Golgi proteins.

## Discussion

Despite the many open questions regarding the mechanism and regulation of Golgi structure and its mitotic inheritance, it is well established that mitotic Golgi disassembly has an active role in cell cycle progression. Two mechanisms converging on the mitotic kinase Aurora-A have been identified. One senses unlinking and activates Src at the GC, resulting in Aurora-A activation to drive entry into mitosis and CE maturation (Barretta et al., 2016). The other is mediated by a GM130-based pathway leading to the activation of TPX2, an allosteric activator of Aurora-A, to control the formation of the spindle astral fibers (Wei et al., 2015; Guo et al., 2021). Furthermore, Golgi-associated structural proteins are repurposed during mitosis to concur to the proper spindle formation (Mascanzoni et al., 2019). Whether additional mechanisms cooperate with the control of entry into mitosis and spindle formation remain to be determined.

Besides, the long-term functional implications of alterations of the mitotic inheritance of the GC are largely unexplored. This is a critical issue as the relocalization of a set of Golgi proteins is necessary for spindle formation (Altan-Bonnet et al., 2003; Mascanzoni et al., 2022). Therefore, errors of GC segregation could result in aberrant spindle formation, which can potentially alter the orientation of cell division, with deleterious consequences on tissue homeostasis, or cause errors of chromosome segregation, favoring cell transformation and tumorigenesis (Matthews et al., 2022). On the other side, premature GC unlinking induces early activation of Aurora-A (Barretta et al., 2016), which can also have important physiological consequences, as untimely Aurora-A activation can reduce the accuracy of the DNA-damage checkpoint, supporting tumorigenesis (Ma and Poon, 2020). Hence, we could speculate that constitutive Golgi unlinking could be a favorable condition for tumorigenesis. Of note, there are reports suggesting that the GC is unlinked in certain cancer cells (Bui et al., 2021) and that GC fragmentation may sustain cell survival and resistance to DNA-damaging agents, limiting the efficacy of chemotherapy (Farber-Katz et al., 2014). Nevertheless, despite the important potential pathological implications of the functional connection between Golgi inheritance and cell division, this topic has been showing a steady decline in interest over the last 15 years, as evidenced by the PubMed search for the words “Golgi” and “mitosis”.

Therefore, we hope for a renewed interest of the scientific community in the investigation of the basic mechanisms and regulation of mitotic Golgi disassembly, its connections with the control of mitotic processes and the effects of alterations of Golgi inheritance on genomic stability. In addition, more systematic studies of the structure and function of the GC in patient tissues and patient-derived cells are much needed for a better understanding of the link between GC and cancer (Bui et al., 2021).

A major challenge is that the Golgi structural proteins are involved in multiple functions, which are probably based on transient and low-affinity associations with the relevant binding partners. Thus, it becomes complicated to identify the direct functional consequences of a specific experimental “perturbation” (e.g., siRNA of a golgin). Finally, we also hope for a raising interest in applying the new available technologies to map transient protein-protein interactions (e.g., BioID, APEX) (Lam et al., 2015) and to selectively perturb in an acute manner selected protein-protein interactions (e.g., inducible expression of point or deletion mutants or rapid, controlled degradation of a single protein) to shed light on the precise structural role of the Golgi matrix proteins and of their interaction with the cytoskeleton, aiming to a broader understating of their regulation by dedicated phospho-proteomic studies.

In our opinion, answering the several open questions discussed in this article could not only address the basic issues of why the GC is in the form of a ribbon and its mitotic partitioning is monitored by a control mechanism, but also lead to the development of new therapeutical applications. As an example, drug-based inhibition of Golgi unlinking could be combined with spindle poisons to selectively induce mitotic catastrophe and death of cancer cells (Serrano-Del Valle et al., 2021). Conversely, cancer cells with a constitutively unliked GC could have an overactivated Aurora-A, thus being more sensitive to DNA damaging agents (Ma and Poon, 2020). These are unexplored possibilities that we think could lead to the development of novel strategies for cancer treatment.

## Data Availability

The original contributions presented in the study are included in the article/Supplementary Material, further inquiries can be directed to the corresponding author.
